# Risk assessment for West Nile Virus in Northern Greece (2010-2013)

**DOI:** 10.1186/1756-3305-7-S1-O2

**Published:** 2014-04-01

**Authors:** S Gewehr, S Kalaitzopoulou, L Slavi, S Mourelatos

**Affiliations:** 1 Ecodevelopment S.A., Thessaloniki, Greece

## 

Since the large WN fever epidemic in Central Macedonia in 2010 caused by WNV Lineage 2, with a total of 262 cases (197 neuroinvasive, incidence rate 1:140), WNV cases are reported every year from different areas throughout Greece (2011: 100 cases/75 neuroinvasive, 2012: 161/109, 2013: 86/51). WN fever has become one of the most important issues for the National Health Authorities in terms of vector-borne diseases.

From 2011 and onwards, for the surveillance of WNV in Central Macedonia, two major networks were established by Ecodevelopment in collaboration with the Hellenic CDC, the region of Central Macedonia and four specialized laboratories: 1) A network of 60 CO_2_-traps for adult mosquitoes at fixed sites monitored biweekly for 4 months every year. Pools of 10-50 *Culex spp*. are forwarded weekly to the laboratories for the detection of WNV. 2) A network of sentinels (domestic pigeons and/or backyard chickens, 40 -50 hencoops or pigeon coops, 400-450 samples/year) for blood sampling in early summer and/or at the end of the hot season.

In the plain of Thessaloniki, in early summer (June) the seroconversion in chickens reached 11,9% (28 positive/236 chickens) in 2011 versus 4,1% (8 positive/197 chickens) in 2013. The corresponding average weekly Minimum Infection Rate (M.I.R.) for the period mid June-end August was 1.73 in 2011 and 0.39 in 2013 respectively. These data seem to support the hypothesis that it is possible to relate the level of WNV circulation (infected mosquitoes and animal sentinels) with the upcoming human WNV cases: 16 human neuroinvasive cases were recorded in 2011 versus 5 cases in 2013.

The follow up of the epidemiological risk through these two networks in combination with the weekly epidemiological reports of the Hellenic CDC and the results of larval habitat monitoring are permanently used to optimize vector control measures that are implemented in the region of Central Macedonia.

Funding: Ecodevelopment, Hellenic Center for Disease Control & Prevention, Integrated Surveillance and Control Programme for West Nile Virus and Malaria in Greece

